# Supplementary material for: A set of multi-entry identification keys to African frugivorous flies (Diptera, Tephritidae)
Source: Zookeys. 2014 Jul 24;(428):97–108. doi: 10.3897/zookeys.428.7366 (PMC4143993; doi:10.3897/zookeys.428.7366)
Supplement: Supplementary material 6 — Key to Ceratitis [file zookeys-428-097-s006.zip › SF6_ZooKeys_key to Ceratitis/key/SF6_ZooKeys_key to Ceratitis/Media/Html/Ceratitis marriotti .htm]

Ceratitis (Ceratalaspis) marriotti Munro


***Ceratitis*** **(*Ceratalaspis*)
*marriotti* Munro**

*Ceratitis* (*Pardalaspis*) *marriotti*
Munro, 1933a: 36.

Body
length: 5.12 (4.60-5.25) mm; wing length: 5.02 (4.75-5.25) mm.

Male

Head.
Antenna yellow. Third antennal segment twice as long as second segment. Arista
with short hairs over entire length. Frons with short scattered hairs which are
usually darker than frons, sometimes paler and almost same colour as frons;
convex, in lateral view not distinctly projecting forwards at antennal implant.
Lower eye margin with darker marking (not always very distinct). Chaetotaxy
normal for subgenus.

Thorax.
Postpronotum white, unspotted. Ground colour of mesonotum pale with orange
tinge; mesonotal pattern, median spot between postpronota variable, sometimes
only present as a darker yellow patch. Chaetotaxy normal for subgenus. Scapular
setae black. One anepisternal bristle. Scutellum white with three black
separate markings apically, these markings hardly visible in dorsal view,
restricted to apical margin; basally without dark spots, only slight darker
colouration. Subscutellum completely dark.

Legs
yellow; setation typical for subgenus, mixed darkish and pale. Posterior and
posterodorsal rows pale, sometimes dark posteriorly.

Wing
bands with yellow-brown markings, sometimes more extensively yellow. Banding,
marginal band with clear interruption after cell R1; cubital band free; medial
band joined with marginal band; crossvein r-m at or before middle of discal
cell. Crossvein dm-cu posteriorly more inwards than anteriorly.

Abdomen.
Yellow (sometimes brownish discolored). Setation and banding typical for
subgenus.

Female

As
male except for the following characters: Frons with scattered hairs that are
always darker than frons. Oviscape shorter than abdominal terga 3-6 combined.

(Description
after De Meyer, 1998)
